# Various green manure-fertilizer combinations affect the soil microbial community and function in immature red soil

**DOI:** 10.3389/fmicb.2023.1255056

**Published:** 2023-12-14

**Authors:** Jing Xu, Linlin Si, Xian Zhang, Kai Cao, Jianhong Wang

**Affiliations:** Institute of Environment, Resource, Soil and Fertilizer, Zhejiang Academy of Agricultural Sciences, Hangzhou, China

**Keywords:** enzyme activity, fertilizer, green manure, immature red soil, soil microbial community

## Abstract

Green manure application is a common practice to improve soil fertility in China. However, the impact of different green manure-fertilizer combinations on the soil microbial communities in the low-fertility immature red soil in southern China remains unclear. In this study, we conducted a pot experiment using two common green manure crops, ryegrass (*Lolium perenne* L.) and Chinese milk vetch (*Astragalus sinicus* L.), along with a fallow treatment. We also considered three combined fertilizer management strategies, including mineral, humic acid, and organic manure fertilizers. We evaluated the soil microbial biomass, activity, communities, functional prediction and their correlation with soil properties during green manure growth and incorporation periods, to assess the potential alterations caused by different green manure and fertilizer combinations. Our findings indicate that green manure application, particularly in combination with organic fertilizers, increased the alpha diversity of the soil bacterial community, while the opposite trend was observed in the fungal community. The application of green manure altered the soil microbial communities during both growth and incorporation periods, especially the taxa that participate in carbon, nitrogen and sulfur cycles. Notably, ryegrass significantly increased the relative abundance of bacterial phylum *Firmicutes* and fungal phylum *Ascomycota*, whereas Chinese milk vetch significantly stimulated the bacterial phylum *Acidobacteria* and fungal phylum *Glomeromycota*. Compared with fallow treatments, green manure application significantly increased the soil pH by 4.1%–12.4%, and microbial biomass carbon by 29.8%–72.9%, regardless of the types of combined fertilizer. Additionally, the application of green manure resulted in a 35.6%–142.6% increase in urease activity and a 65.9%–172.9% increase in *β*-glucosidase activity compared to fallow treatments, while led to a 22.5%–55.6% decrease in catalase activity. Further analysis revealed that the changes in both bacterial and fungal communities positively correlated with soil pH, soil organic matter, total nitrogen and alkali hydrolyzed nitrogen contents. Moreover, the relationship between the soil microbial community and soil enzyme activities was regulated by the specific green manure species. In conclusion, our results provide insight into the effects of different green manure-fertilizer combinations on soil microorganisms and their underlying mechanisms in improving soil fertility in the low-fertility immature red soil.

## 1 Introduction

As large-scale farming expands in the red soil of hilly regions in southern China, some immature red soil has been reclaimed as farmed topsoil. However, the low levels of organic matter, nutrients, and biological activity in immature red soil limit crop yields (Xu et al., 2003; Wilson et al., 2004; Bao et al., 2019). Thus, improving soil fertility is crucial in immature red soil fields. Green manure and fertilization can affect soil carbon sequestration, plant growth, and microbial community structure (Elfstrand et al., 2007; Chavarría et al., 2016; Maitra et al., 2018). Green manure application is a traditional farming practice in China, which assists in creating sustainable agricultural systems and plays an important role in maintaining fertility and crop productivity (Tejada et al., 2008a; Pittelkow et al., 2015). Previous studies have demonstrated that green manure crops can improve soil physical and chemical properties by forming and stabilizing soil macroaggregates and increasing soil organic matter and nutrient availability (Jama et al., 2000; Hwang et al., 2015; Adetunji et al., 2020). Furthermore, the application of green manure can influence soil biological properties, such as the microbial activity, diversity and communities in agricultural soils (Caban et al., 2018; Gao et al., 2020; He et al., 2020). Thus, the application of green manure as one major organic material to increase organic inputs to rice production systems is considered as the most practical option to enhance the organic matter dynamics and nutrient cycling in low-fertility immature red soil.

Soil microorganisms are the key to ecological processes such as soil organic matter decomposition, nutrient cycling, and other critical agroecosystem processes that promote plant growth and soil quality (Bardgett and van der Putten, 2014; Fierer, 2017). Green manure plants can regulate their rhizosphere microbiome through rhizodeposition and the nutrient trade-off between soil and plants during the growth period (Gleixner et al., 2002; Pausch and Kuzyakov, 2018; Saleem et al., 2020). Moreover, the incorporation of green manure crops into the soil provides fresh organic matter for microorganisms, which benefits soil by increasing the soil microbial biomass and enhancing enzyme activities in agricultural systems (Tejada et al., 2008b; Hwang et al., 2015; Khan et al., 2019; Brtnicky et al., 2021). As soil enzymes are mainly released from soil microorganisms, enzyme activities can indicate potential changes in the soil microbial communities and biochemical processes (Sinsabaugh et al., 2008). The activities of soil enzymes such as catalase, *β*-glucosidase, and urease are associated with the degradation of organic matter and the cycling of carbon and nitrogen (Stott et al., 2010; Dharmakeerthi and Thenabadu, 2013; Kolesnikov et al., 2019). Zheng et al. (2018a) found that green manure incorporation significantly enhanced the activities of *β*-glucosidase, *β*-xylosidase, and cellobiohydrolase, which can accelerate green manure degradation, and this was correlated with relative increases in the families *Ruminococcaceae* and *Lachnospiraceae*. The effects of green manure on different enzyme activities are reported to vary with the species of green manure, suggesting that specific dominant microbes are established when different green manure species are applied to the soil (Taylor et al., 2002; Gao et al., 2016; Khan et al., 2019). Recently, high-throughput sequencing has been used to assess the effects of green manures on soil microbes, and these studies have reported that the microbial community composition changes according to the types (e.g., leguminous and non-leguminous) of green manure crops used (Kataoka et al., 2017; Tao et al., 2017; Zhang et al., 2017, 2020; Silva et al., 2021). With regard to newly cultivated red soil with low fertility, however, research on the effects of different types of green manure on soil biological properties, especially microbial communities, during both green manure growth and incorporation, is limited.

Because of the low levels of organic matter and available nutrients in immature red soil, other fertilizers such as mineral fertilizer, biochar, pig manure, and humic compounds are usually applied together with green manure to accelerate soil quality improvement (Marschner et al., 2003; Katterer et al., 2019; Han et al., 2021). Previous studies showed that the application of green manure combined with different fertilizers has various effects on soil properties, altering the microbial biomass, activity, and community structure (Baumann et al., 2009; Lori et al., 2017). Green manure in combination with mineral fertilizer has been suggested to be more effective at increasing soil carbon stability and grain yields than using either mineral fertilizer or green manure alone (Gao et al., 2016; Das et al., 2019; Kamran et al., 2021). A recent study showed that green manure (*Arachis pintoi*) combined with natural phosphate promoted enzyme activities and affected the soil microbial community structure (de Medeiros et al., 2019). In addition, humic compounds, such as humic acid, play a role in a variety of soil processes and are related to soil physical and chemical properties, influencing the soil nutrient cycle and nutrient bioavailability (Moran et al., 2005; Olk et al., 2019). Also, organic manure, produced by composting and fermenting livestock manure, is usually used to improve soil fertility in agricultural systems (Lu et al., 2011). In a previous study, we found that both humic acid and organic manure improved soil fertility, and the combined application of organic amendments and mineral fertilizer maximized economic benefits by increasing rice grain yield and enhancing soil quality, resulting in a profit increase of 17%–33% (Si et al., 2023). Therefore, these two organic fertilizers are commonly utilized in practice. Previous studies have indicated the positive effects of combining green manure with organic fertilizer on soil humus and soil organic carbon accumulation in long-term agricultural systems (Ghosh et al., 2018; Wen et al., 2018; Masilionyte et al., 2021). However, the impact of various combinations of green manure and fertilizers on the characteristics and function of microbial community at the initial stage of low-fertility soil cultivation remains unclear. Understanding these factors is crucial for gaining further insights into how green manure practices influence soil fertility and health in the immature red soil.

Ryegrass (*Lolium perenne* L.) and Chinese milk vetch (*Astragalus sinicus* L.) emerge as the primary green manure variations in southern China, extensively cultivated in green manure-rice rotation systems in preference to winter fallow (Lei et al., 2022). Ryegrass, belonging to the *Poaceae* family, exhibits a robust root system and displays strong adaptability to low-quality soils, resulting in substantial biomass production (He et al., 2020). Chinese milk vetch is considered as the most popular green manure in southern China since its higher nitrogen fixing capability. Consequently, the residues of ryegrass have a higher C:N ratio than Chinese milk vetch. The differences in plant composition and ecological functions of the two green manure species may result in varying impacts on soil properties during both growth and incorporation periods. Overall, the goal of the study was to estimate the effects of two types of green manure, ryegrass and Chinese milk vetch, in combination with mineral fertilizer, humic acid, or organic manure on the characteristics of the soil microbial community. This included evaluating the growth, activity, composition, and function of the microbial community in immature red soil. To control the environmental conditions, a greenhouse pot experiment was conducted to test the following hypotheses: (a) the growth and incorporation of green manure have the potential to stimulate specific functional groups of the soil microbial community that are involved in nutrient cycling; (b) the application of various green manure-fertilizer combinations may improve the soil physicochemical and biological properties of low-fertility immature red soil by altering the microbial community; and (c) these effects on the soil microbial community are contingent upon the specific green manure plants species utilized.

## 2 Materials and methods

### 2.1 Study site and soil characteristics

The experiment was carried out in a greenhouse in western Zhejiang Province, China (119° 3′ 26′′ E, 28° 56′ 06′′ N), where the soil parent material comprises calcareous red sandstone, sand shale, and red glutenite and has a weak structure. The immature red soil used for the experiment was collected randomly from red subsoil in a hilly area in Quzhou, Zhejiang Province. The basic physical and chemical properties of the soil were as follows: pH (1:5, soil:water) 5.30, soil organic matter 2.51 g kg^−1^, total nitrogen (N) 0.28 g kg^−1^, total phosphorus 0.68 g kg^−1^, total potassium 3.6 g kg^−1^, alkali hydrolyzable nitrogen 21.2 mg kg^−1^, available phosphorus 3.05 mg kg^−1^, and available potassium 55 mg kg^−1^.

### 2.2 Experimental design and sampling

**Figure 1:**
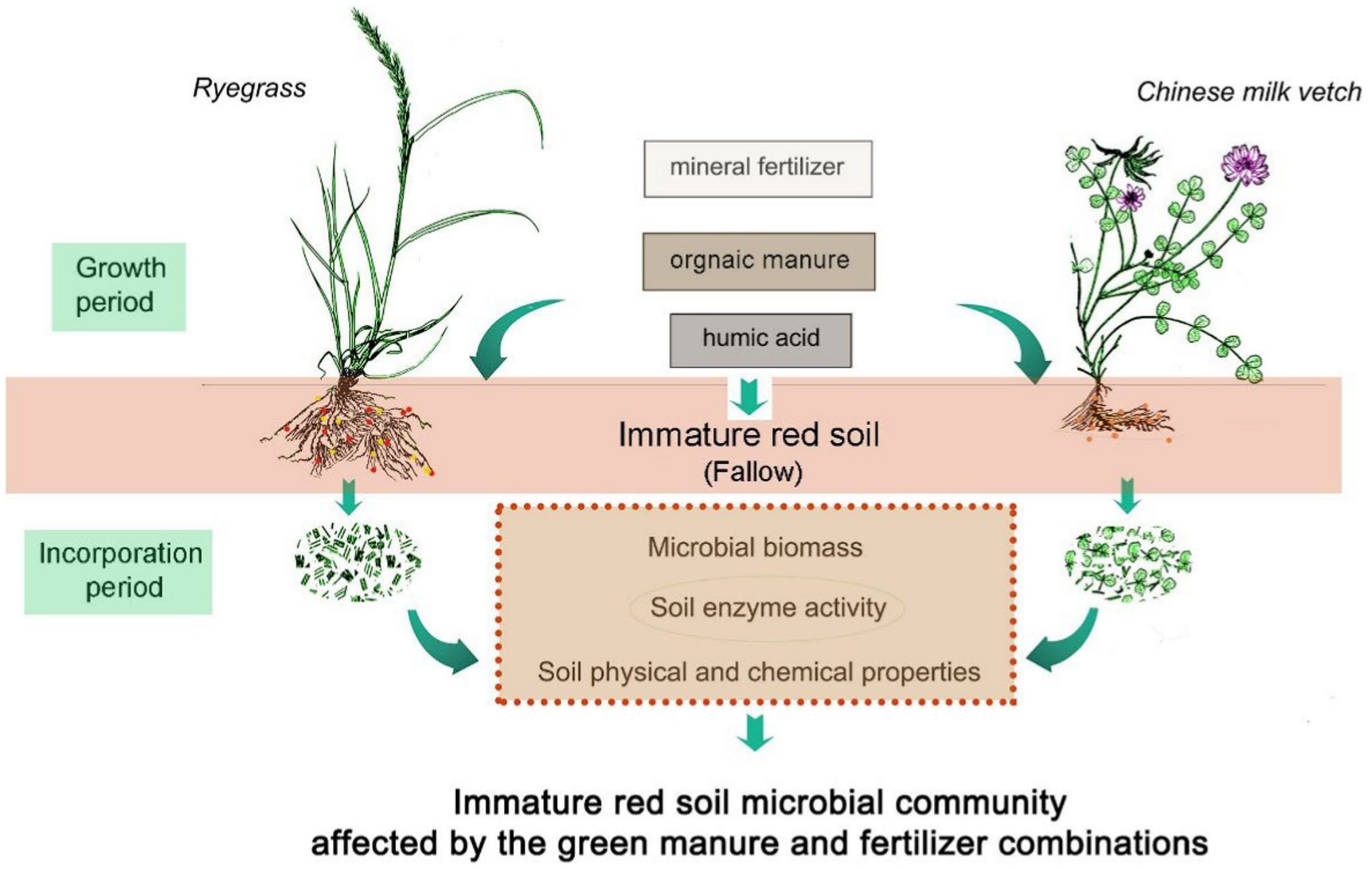
Diagram of the experimental design. The fallow treatment was the soil without green manure application, while the ryegrass and Chinese milk vetch application treatments including growth period and incorporation period. All the three treatments were combined with three fertilizers, including mineral fertilizer, organic manure and humic acid fertilizer. Also, a control without green manure and fertilizer was set up.

Two types of green manure application, ryegrass or Chinese milk vetch, were compared to a fallow treatment, in combination with three types of fertilizers, including mineral fertilizer, humic acid fertilizer, or organic manure. A control treatment without green manure plants or fertilizer was also included. As a result, a total of 10 treatments were designed as follows: control treatment (CK); fallow soil with mineral fertilizer (CF), fallow soil with humic acid fertilizer (HAF), fallow soil with organic manure (OMF), ryegrass combined with mineral fertilizer (RG), ryegrass combined with humic acid fertilizer (RH), ryegrass combined with organic manure (RO), Chinese milk vetch combined with mineral fertilizer (MG), Chinese milk vetch combined with humic acid fertilizer (MH), and Chinese milk vetch combined with organic manure (MO). Each treatment was replicated three times (Figure 1). The organic matter contents of the humic acid fertilizer (Ruiyu Trading Co. Ltd., Shandong, China) and the organic manure (Fengji Biotech Co., Ltd., Jiangxi, China) are 75.3% and 29.2%, respectively. The nutritional content details are shown in Supplementary Table S1.

Each pot (volume 10.1 L, height 31 cm) held 10.0 kg of red soil (DW) screened through 5 mm meshes. Except for the CK, all treatments received mineral fertilizer (N: 0.05 g kg^−1^ urea, P: 0.12 g kg^−1^ Ca(H_2_PO_4_)_2_ and K: 0.04 g kg^−1^ KCl) prior to green manure plants seeding. The humic acid or organic manure was thoroughly mixed with the potted soil of HAF, RH and MH or OMF, RO and MO, respectively, to achieve a concentration of 20 g kg^−1^ (WW). Before sowing, seeds were disinfected and rinsed with 5% sodium hypochlorite. Seedlings were thinned to 15 seedlings per pot after germination. Supplementary water was added as needed during plant growth.

The study began in November 2018. The average temperatures during the growth period ranged from 7.2°C to 15.6°C and during the incorporation period from 16.5°C to 25.0°C. After 5 months of growth, the green manure crops were harvested at the beginning of the reproductive growth stage. For the six treatments that involved planting ryegrass and Chinese milk vetch, the shoots of the green manure crops were cut and the roots carefully separated from the soil. To collect the rhizosphere soil adhering to the roots of the green manure crops, we gently shook the soil from the roots located in the topsoil layer (2–10 cm; Nazih et al., 2001; Wang et al., 2009). In order to maintain consistent sampling depth, for the three fallow treatments and CK, we sampled the topsoil (2–10 cm) using a 38 mm diameter soil core sampler. The soil samples were collected in sterile tubes, transported to the laboratory under liquid nitrogen, and stored at −80°C until further analysis.

The fresh shoots of ryegrass and Chinese milk vetch in each treatment were cut chopped into approximately 3 cm lengths and thoroughly mixed within each set of three replicates. Then, precisely weighed 200.0 g fresh residues of each treatment and evenly incorporated into the soil of the respective pot. During the incorporation period, the soil was maintained in a flooded state for 8 weeks, according to field practices, and the soil was sampled in May 2019. Throughout the study, no chemical plant protection agents (such as fungicides, pesticides, or herbicides) were used. From all 10 treatments, approximately 500 g soil samples were removed using a 38 mm diameter soil core sampler at the depth of 0–15 cm from each pot and divided into three parts. The first subsample was collected in sterile tubes (50 mL) and stored at −80°C prior to total soil DNA extraction. The second sub-sample was sieved through a 2 mm sieve and stored at 4°C to determine the soil microbial biomass carbon and enzyme activity within 7 days. The last subsample was air-dried and finely ground to pass through 2 and 0.20 mm sieves prior to analyzing its physical and chemical properties.

### 2.3 Samples analyses

To determine the soil microbial biomass carbon, chloroform fumigation and K_2_SO_4_ extraction were performed, and the extract was analyzed with a carbon and nitrogen analyzer (Vance et al., 1987). The activity of *β*-glucosidase was determined using the *p*-nitrophenol colorimetric method (Eivazi and Tabatabai, 1988). Urease activity and catalase activity were measured using the phenol-sodium hypochlorite colorimetric method and the potassium permanganate titration method, respectively, following the protocols described by Guan et al. (1986). Soil pH was measured using a pH meter (Mettler-Toledo FE20, Shanghai, China) in a soil and distilled water suspension (1:5, w/v). Soil organic matter (SOM), total nitrogen content (TN), total phosphorus content (TP), Olsen-phosphorus content (OP), and alkali hydrolyzale nitrogen content (AN) were measured according to the methods described by Lu (1999) with some modifications. Specifically, SOM was measured using the potassium dichromate external heating method. TN was analyzed using the SKD-100 automated Kjeldahl nitrogen analyzer (PEIOU, Shanghai, China). AN was determined using a reduction diffusing method after alkaline hydrolysis. TP and OP were measured using extraction and colorimetric methods.

A kit (MP FastDNA® Spin Kit for Soil) was used to extract total DNA from the soil. The quality of DNA was assessed by 1% agarose gel electrophoresis. The V3-V4 hypervariable region of the 16S rRNA gene was selected for PCR amplification with primers 338F (5′-ACT CCT ACG GGG AGG CAG CAG-3′) and 806R (5′-GGA CTA CHV GGG TWT CTA AT-3′). The ITS1-ITS2 region of the fungal gene was amplified with primers ITS1F (5′-CTT GGT CAT TTA GAG GAA GTA A-3′) and ITS2 (5′-GCT GCG TTC TTC ATC GAT GC-3′). The PCR reactions were conducted by using the programs: 3 min of denaturation at 95°C; followed by 27 cycles of 30 s at 95°C, 30 s at 55°C, and 45 s at 72°C; and a final extension at 72°C for 10 min with a thermocycler PCR system (GeneAmp 9700, ABI, United States). Sequencing for bacterial and fungal communities was accomplished using an Illumina MiSeq PE300 platform by the Majorbio Company (Shanghai, China), following the manufacturer’s instructions. All the raw sequences tested in the study were uploaded to the NCBI SRA database (accession nos., PRJNA992634 and PRJNA992506).

### 2.4 Statistical analysis

SPSS software (v.21.0) was used for statistical analyses to test the effects of green manure and fertilizer types on soil properties, microbial biomass carbon, and enzyme activities with two-way ANOVA and to compare the differences between treatments with one-way ANOVA. The data were compared by the least significant difference (LSD) method at the 5% confidence level. Levene’s test was used to check the homogeneity of variance.

The sequencing data of soil bacterial and fungal communities from green manure growth and incorporation periods were analyzed as described by Xu et al. (2018). Briefly, the original sequencing data were quality-filtered, merged, and optimized; then, the Operational Taxonomic Units (OTUs) with 97% similarity were clustered by using the UPARSE pipeline after removing the chimeric sequences (Edgar, 2013). The taxonomy of the bacterial and fungal OTUs was analyzed against the Sliva (SSU138) 16S rRNA database and Unite (Release 8.0) database, respectively.

The alpha diversity was assessed according to the Chao1 and Shannon indices, which indicated the richness and diversity of the microbial community, respectively. Principal coordination analysis (PCoA) based on the OTU level was used to determine the degree of dissimilarity among bacterial and fungal communities in the 10 treatments. Redundancy analysis (RDA) or canonical correspondence analysis (CCA) at the genus level were performed to investigate the relationships between the bacterial and fungal communities of soil samples from each treatment and soil enzyme activities, respectively. And the distance-based RDA (db-RDA) at the bacterial and fungal genus level were performed to investigate the relationships between the soil samples from each treatment and the soil physical and chemical properties. Analysis of similarities (ANOSIM) was used to assess the statistical significance between different treatments. Comparisons of two groups of bacterial or fungal taxa were analyzed with Welch’s *t*-test. A multiple groups comparison of bacterial or fungal taxa and linear discriminant analysis (LDA) effect size (LEfSe)^1^ were used to elucidate the significant differences in bacterial or fungal taxa among the green manure treatments (ryegrass and Chinese milk vetch) and fallow treatment, regardless the combined fertilizers. The functional groups of the bacterial community were predicted using the functional annotation of prokaryotic taxa (FAPROTAX) database^2^ based on the 16S rRNA gene data (Louca et al., 2016). And functional properties of the fungal community were assigned using the FUNGuild database^3^ based on the ITS gene data (Nguyen et al., 2016). The data were analyzed using a combination of R packages, the Galaxy web application, and the functional prediction tools mentioned above, all of which were accessed through the Majorbio Cloud Platform (Ren et al., 2022).

## 3 Results

### 3.1 Effects of green manure growth and fertilizer combinations on the rhizosphere soil microbial community

#### 3.1.1 Soil microbial community composition

**Figure 2:**
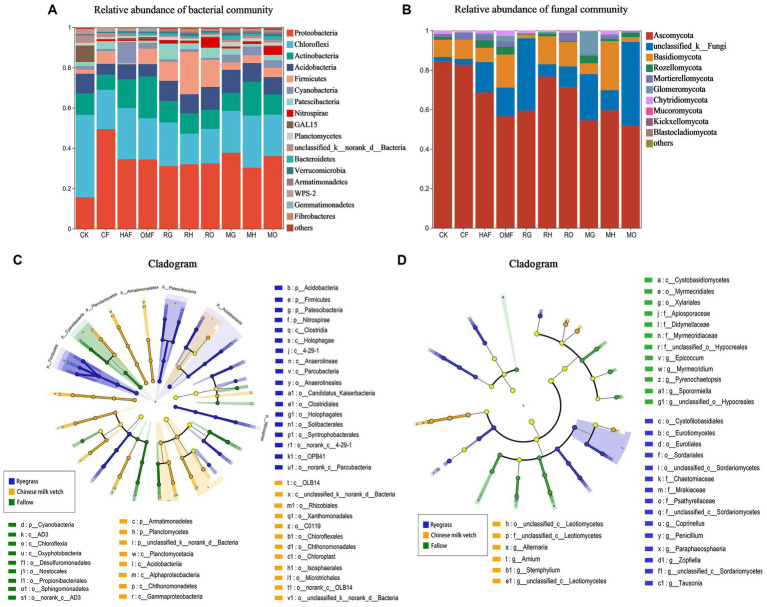
The relative abundance of **(A)** bacterial community composition and **(B)** fungal community composition at phylum level in bulk soil of fallow treatments and rhizosphere soil during green manure growth period. Regardless of the combined fertilizers, the LEfSe of **(C)** bacterial community and **(D)** fungal community in bulk soil of fallow treatments and rhizosphere soil during green manure growth period. Cladograms indicate the phylogenetic distribution of microbial lineages associated with the fallow, ryegrass and Chinese milk vetch treatments. The most different bacteria are represented in the different color. Each circle’s diameter is proportional to the taxon’s abundance. The blue, yellow, and green taxa indicate the biomarkers in ryegrass, Chinese milk vetch and fallow treatments, respectively.

From the soil samples taken during the green manure growth period, approximately 1,472,105 effective bacterial sequences and 1,830,116 fungal ITS region sequences were obtained, respectively. A total of 4,564 bacterial OTUs and 3,017 fungal OTUs were obtained at 97% similarity. Among 38 bacterial phyla, the most abundant phylum in all treatments except for the CK was *Proteobacteria*, accounting for 30.32%–49.41% of the total abundance, followed by *Chloroflexi* (15.16%–25.90%), *Actinobacteria* (7.52%–20.78%), *Acidobacteria* (5.37%–11.38%), and *Firmicutes* (0.74%–20.99%; Figure 2A). Both ryegrass and Chinese milk vetch stimulated the relative abundance of *Acidobacteria*. The relative abundance of *Firmicutes* in ryegrass rhizosphere soil was 3.6- and 3.0-fold higher than in Chinese milk vetch rhizosphere soil and fallow soil, respectively (Supplementary Figure S1). With respect to the different fertilizer types, the relative abundance of *Firmicutes* was more enhanced in RH than in RG (*p* < 0.01) and RO (*p* < 0.05), while there were no significant differences at the phylum level between the three types of fertilizers in the rhizosphere soil of Chinese milk vetch (*p* > 0.05). Among the 14 fungal phyla, the most abundant phylum was *Ascomycota* (51.82%–84.43%), in which the relative abundance of the classes *Sordariomycetes* and *Dothideomycetes* were significantly lower in the ryegrass rhizosphere than in fallow soil when combined with mineral fertilizer and organic manure, respectively (Figure 2B; Supplementary Figure S2).

In addition, LEfSe (LDA > 3.5) detected bacterial and fungal biomarkers in the rhizosphere soil of green manure. The bacterial phylum *Nitrospirae* (LDA = 3.94, *p* < 0.05) was significantly affected by planting ryegrass, and the order *Rhizobiales* (LDA = 4.02, *p* < 0.01) was significantly stimulated by Chinese milk vetch (Figure 2C). The fungal orders *Eurotiales* and *Sordaridles* in the ryegrass rhizosphere soil were significantly different from those in the Chinese milk vetch rhizosphere soil and fallow soil (Figure 2D).

#### 3.1.2 Soil microbial alpha diversity

The soil bacterial richness and diversity, as indicated by the Chao1 and the Shannon indices, were enhanced in the rhizosphere soil of both types of green manure when combined with the mineral fertilizer (Table 1). Compared with the *CF* treatment, the Chao 1 exhibited a 39.7% increase in the RG treatment and a 60.0% increase in the MG treatment. Similarly, the Shannon indices showed a 21.4% increase in the RG treatment and a 28.2% increase in the MG treatment. When combined with humic acid fertilizer, the Shannon indices in the RH and MH treatments were 8.8% and 9.6% higher, respectively, than those in the HAF treatment. Although the Chao1 and the Shannon indices were highest in the MO treatment among all treatments, there were no significant differences among the green manure treatments and fallow treatments when combined with organic manure (OMF vs. RO and MO).

**Table 1:** Bacterial and fungal diversity indices in rhizosphere soil of green manure treatments and bulk soil of fallow treatments during growth period, and in the bulk soil of green manure treatments and fallow treatments during incorporation period.

Treatment combination	Growth period				Incorporation period			
	Bacterial community		Fungal community		Bacterial community		Fungal community	
	Chao 1	Shannon	Chao 1	Shannon	Chao 1	Shannon	Chao 1	Shannon
CK	1177.63 ± 14.21a	4.73 ± 0.07a	562.88 ± 43.02abc	3.16 ± 0.19ab	1276.86 ± 23.83ab	4.67 ± 0.12a	259.70 ± 21.09ab	3.59 ± 0.22bc
CF	1100.39 ± 72.99a	4.44 ± 0.07a	387.74 ± 41.96abc	3.40 ± 0.45abc	1159.02 ± 82.84a	4.83 ± 0.18ab	218.23 ± 60.60a	1.86 ± 0.60a
HAF	1745.05 ± 138.59bc	5.22 ± 0.23b	714.37 ± 83.31cd	3.94 ± 0.23c	1498.00 ± 140.14bc	4.99 ± 0.18ab	397.50 ± 76.75b	3.51 ± 0.52bc
OMF	2016.07 ± 90.93de	5.56 ± 0.03cd	761.41 ± 54.29d	3.98 ± 0.20c	1666.10 ± 70.43cd	5.05 ± 0.05bc	365.09 ± 106.31ab	3.75 ± 0.17c
RG	1537.64 ± 73.17b	5.39 ± 0.11bc	360.02 ± 49.90a	2.80 ± 0.34a	1609.67 ± 53.09cd	5.34 ± 0.10cd	386.20 ± 37.92b	2.63 ± 0.38ab
RH	1837.31 ± 42.01cd	5.68 ± 0.07cd	405.73 ± 78.98ab	3.66 ± 0.32bc	1651.04 ± 63.79cd	5.34 ± 0.12cd	362.60 ± 36.34ab	2.84 ± 0.16abc
RO	2005.49 ± 129.75cde	5.59 ± 0.18cd	528.23 ± 104.03abc	3.73 ± 0.09bc	2061.47 ± 59.38e	5.79 ± 0.06e	417.14 ± 58.84b	2.92 ± 0.23bc
MG	1760.00 ± 98.62cd	5.69 ± 0.10cd	572.89 ± 96.16bc	3.69 ± 0.11bc	1605.48 ± 140.64cd	5.42 ± 0.13d	413.68 ± 32.73b	2.80 ± 0.36abc
MH	1978.24 ± 36.05cde	5.72 ± 0.09cd	497.93 ± 56.90abc	3.38 ± 0.22abc	1855.25 ± 23.49de	5.57 ± 0.04de	368.42 ± 2.57ab	2.47 ± 0.29a
MO	2225.82 ± 100.31e	5.76 ± 0.06d	527.30 ± 53.54abc	2.66 ± 0.25a	1839.13 ± 106.18de	5.56 ± 0.08de	476.39 ± 58.12b	2.62 ± 0.21ab

Chao 1 index showed the richness of the soil microbial community and Shannon index showed the diversity of the soil microbial community. Values are means ± SE of three replicates. Means in column followed by the same letter are not significantly different (*p* > 0.05). CK, the control treatment; CF, fallow soil with mineral fertilizer; HAF, fallow soil with humic acid fertilizer; OMF, fallow soil with organic manure; RG, ryegrass combined with mineral fertilizer; RH, ryegrass combined with humic acid fertilizer; RO, ryegrass combined with organic manure; MG, Chinese milk vetch combined with mineral fertilizer; MH, Chinese milk vetch combined with humic acid fertilizer; MO, Chinese milk vetch combined with organic manure.

In terms of the soil fungal community, both the Chao1 and the Shannon indices were significantly lower in the RG treatment compared to the MG treatment (Table 1). In addition, the Chao1 in the RO treatment and the MO treatment were 30.6% and 30.7% lower than that in the OMF treatment, respectively. Furthermore, the Shannon index in the MO showed a 33.2% reduction compared to the OMF treatment.

#### 3.1.3 Soil microbial community structure

**Figure 3:**
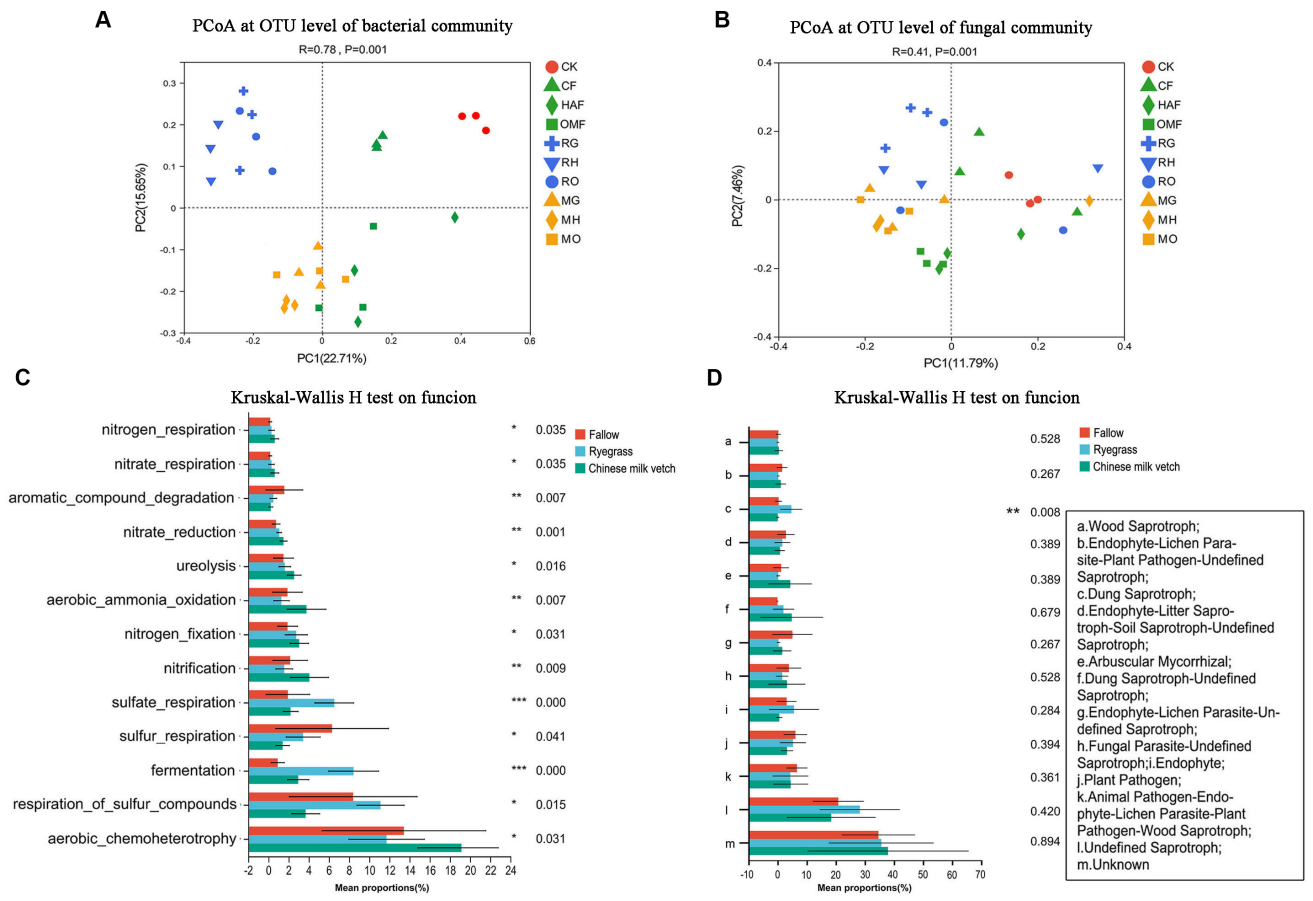
PCoA analysis at OTU level of **(A)** soil bacterial community and **(B)** fungal community in green manure growth period. Samples of CK are shown as red circles. The fallow treatments, ryegrass incorporation treatments and Chinese milk vetch incorporation treatments are shown as green, blue and orange symbols, respectively. The combined mineral fertilizer, humic acid fertilizer and organic manure are indicated by triangle, diamond and square, respectively. Regardless of the combined fertilizers, the functional prediction of **(C)** bacterial community by FAPROTAX and **(D)** fungal community by FUNGuild analysis. The fallow, ryegrass and Chinese milk vetch treatments are shown as red, blue and green bars. Values are means ± SE of three replicates. The ^*^, ^**^, and ^***^ was indicates the *P* < 0.05, *P* < 0.01, and *P* < 0.001 (Kruskal-Wallis *H*-test), respectively.

According to the PCoA based on the OTU level, the bacterial communities in the rhizosphere soils were clustered according to the green manure species (ANOSIM: R = 0.71, *p* = 0.001) more than combined fertilizer types (ANOSIM: R = 0.35, *p* = 0.001; Figure 3A). While, the fungal communities in the rhizosphere soil of the green manure crops showed weakly significant differences from those in the fallow treatments (ANOSIM: R = 0.208, *p* = 0.001), so was located among the three fertilizer types (ANOSIM: R = 0.120, *p* = 0.009; Figure 3B).

#### 3.1.4 Functional prediction of the microbial community in rhizosphere soil

Through FAPROTAX functional prediction, 66 functions were annotated in bacterial communities, and the prominent functions were related to carbon (C), nitrogen (N) and sulfur (S) cycling and were shown to be significantly affected by the growth of green manure plants (Figure 3C). Among the functional groups involved in C cycling, the relative abundance of aerobic chemoheterotrophy predicted functions was significantly higher in rhizosphere soil of Chinese milk vetch than ryegrass, while the opposite was found for fermentation. The abundant bacterial groups involved in the N cycle such as nitrification, nitrogen fixation, aerobic ammonia oxidation, nitrate reduction, nitrate respiration, nitrogen respiration and ureolysis were significantly higher in rhizosphere soil of Chinese milk vetch than ryegrass and fallow treatments. The abundances of functional groups involved in the S cycle, such as respiration of sulfur compounds, sulfur respiration and sulfate respiration, however, were significantly higher during ryegrass growth than Chinese milk vetch.

FUNGuild analysis predicted 9 trophic mode groups of fungal community, with saprotroph being the major function (Fallow: 22.5%, ryegrass: 38.1% and Chinese milk vetch: 24.2%). The relative abundance of dung saprotroph was significantly higher in rhizosphere soil of ryegrass than Chinese milk vetch and fallow treatments (Figure 3D).

### 3.2 Effects of green manure incorporation and fertilizer combinations on soil properties

#### 3.2.1 Soil physicochemical properties

The application of green manure combined with fertilizers had a significant impact on soil pH, SOM, TN, and AN (Table 2; Supplementary Table S2). Compared to the fallow treatments, the application of ryegrass led to a significant increase in soil pH ranging from 9.1% to 12.4%. Whereas the application of Chinese milk vetch increased soil pH by 4.1% in MG and 5.0% in MH, but no significant increase was observed in MO. Also, the TN contents in both the RG and MG treatments were 14% higher than the *CF* treatment. Additionally, the contents of AN were significantly increased in RG and RO treatments, whereas no statistically significant improvement was observed in the Chinese milk vetch application treatments. In addition, the SOM content showed a remarkable increase when combined with humic acid fertilizer, surpassing that of organic manure in both green manure application and fallow treatments.

**Table 2:** Effects of different green manure and fertilizers combinations on soil properties including pH, soil organic matter (SOM), total nitrogen content (TN), total phosphorus content (TP), Olsen-phosphorus (OP), and alkali-hydrolyzale nitrogen (AN).

	pH	SOM (g kg^−1^)	TN (g kg^−1^)	TP (g kg^−1^)	AN (mg kg^−1^)	OP (mg kg^−1^)
CK	5.04 ± 0.03b	7.55 ± 0.44a	0.46 ± 0.01a	1.04 ± 0.09a	52.87 ± 3.38a	4.27 ± 0.04a
CF	4.83 ± 0.05a	7.60 ± 0.62a	0.50 ± 0.01a	1.09 ± 0.07ab	54.50 ± 1.79ab	4.41 ± 0.21ab
HAF	5.16 ± 0.03b	18.85 ± 1.02c	0.63 ± 0.02c	1.13 ± 0.08abc	62.93 ± 1.46abc	4.37 ± 0.20b
OMF	5.05 ± 0.04 b	10.46 ± 0.72b	0.68 ± 0.01de	1.18 ± 0.03abc	69.37 ± 2.78c	4.48 ± 0.27ab
RG	5.43 ± 0.07c	8.13 ± 0.17a	0.57 ± 0.03b	1.02 ± 0.01a	87.43 ± 3.03d	4.36 ± 0.15ab
RH	5.63 ± 0.04d	20.44 ± 0.18c	0.65 ± 0.01cd	1.12 ± 0.02abc	60.73 ± 6.20abc	4.91 ± 0.05b
RO	5.65 ± 0.06d	10.86 ± 0.48b	0.70 ± 0.01e	1.13 ± 0.01abc	81.73 ± 7.29d	4.41 ± 0.18ab
MG	5.03 ± 0.07b	7.81 ± 0.95a	0.57 ± 0.02b	1.11 ± 0.12ab	65.37 ± 2.68bc	4.84 ± 0.20b
MH	5.42 ± 0.06c	20.65 ± 0.91c	0.67 ± 0.02cde	1.27 ± 0.05c	64.80 ± 5.52abc	4.80 ± 0.19ab
MO	5.17 ± 0.07 b	10.87 ± 0.17 b	0.64 ± 0.01 cd	1.24 ± 0.06bc	65.57 ± 3.39bc	4.71 ± 0.31ab

Values are means ± SE of three replicates. Means in column followed by the same letter are not significantly different (*P* > 0.05). CK, the control treatment; CF, fallow soil with mineral fertilizer; HAF, fallow soil with humic acid fertilizer; OMF, fallow soil with organic manure; RG, ryegrass combined with mineral fertilizer; RH, ryegrass combined with humic acid fertilizer; RO, ryegrass combined with organic manure; MG, Chinese milk vetch combined with mineral fertilizer; MH, Chinese milk vetch combined with humic acid fertilizer; MO, Chinese milk vetch combined with organic manure.

#### 3.2.2 Soil microbial biomass carbon content

**Figure 4:**
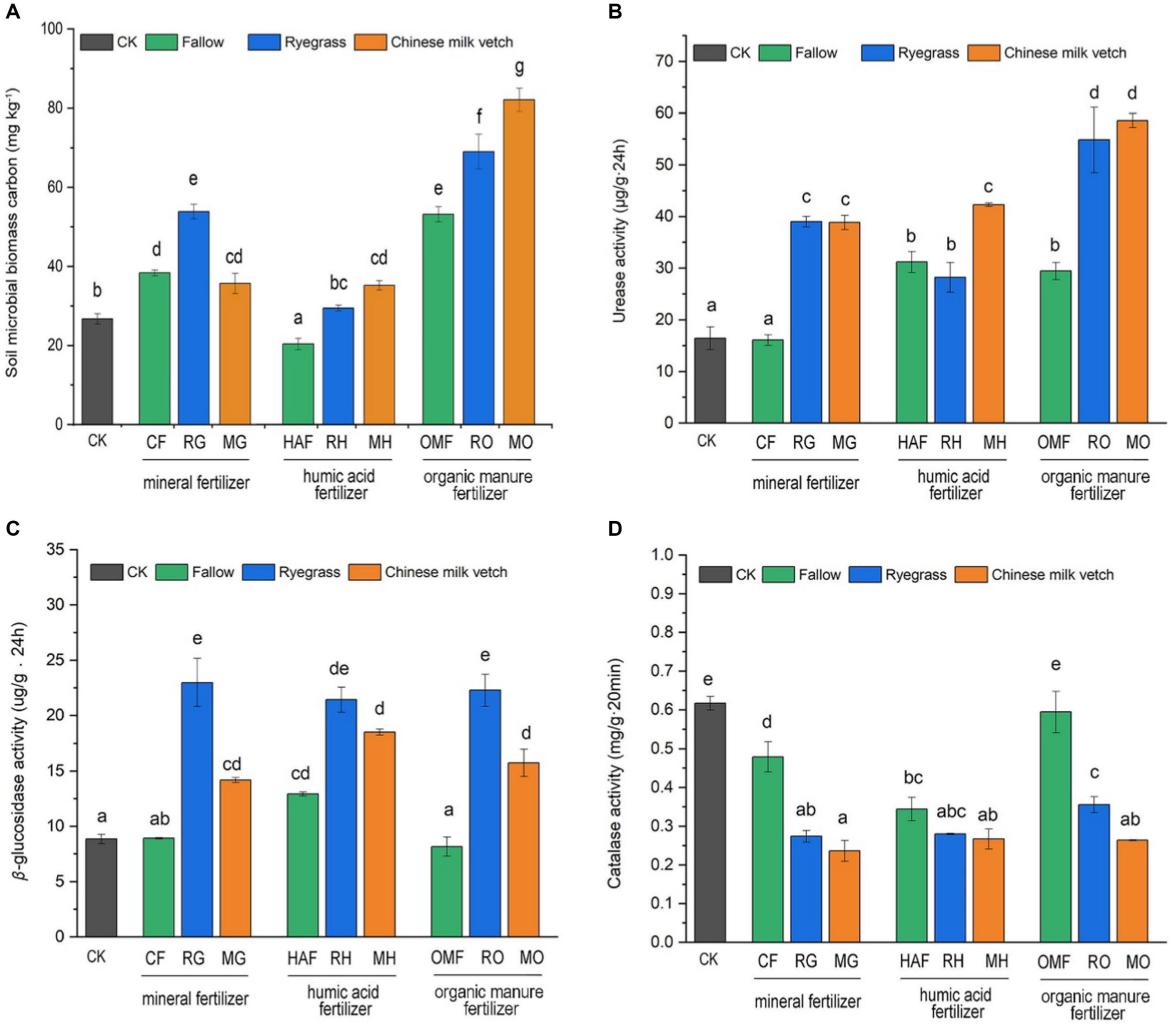
Effects of application of green manure combined with different fertilizers on **(A)** the soil microbial carbon, **(B)** urease activity, **(C)** *β*-glucosidase activity and **(D)** catalase activity during the green manure incorporation period. The CK is shown as the gray bar, and the fallow treatments, ryegrass incorporation treatments and Chinese milk vetch incorporation treatments are shown as green, blue and orange bars, respectively. Different lowercase letters indicate significant differences (*p* < 0.05).

Both green manure incorporation (F_2,27_ = 31.98, *p* < 0.001), combined fertilizer application (F_2,27_ = 243.16, *p* < 0.001), and their interaction (F_4,27_ = 24.19, *p* < 0.001) significantly affected the soil microbial biomass carbon content (SMBC). Compared with CF, the SMBC showed a 40.6% increase in RG, but not in MG. While the SMBC in MO treatment exhibited the highest value among the 10 treatments. The SMBC in RH and MH treatments were 44.7% and 72.9% higher, respectively, compared to the HAF treatments. However, the SMBC was significantly decreased by humic acid fertilizer, compared to the organic manure (Figure 4A).

#### 3.2.3 Soil enzyme activities

Compared with the fallow treatments, green manure application significantly increased soil urease and *β*-glucosidase activities but inhibited catalase activity when combined with the mineral fertilizer and organic manure (Figures 4B–D). Under the humic acid fertilizer treatments, however, no significant differences in the activity of these three enzymes were seen between the fallow and green manure application treatments, except that urease activity in MH was higher than in HAF. The two green manure species also had different effects on enzyme activities. Urease activity was significantly higher in MH than in RH, while *β*-glucosidase and catalase activities were 1.41- and 1.38-fold higher in RO than in MO, respectively.

### 3.3 Soil microbial community during green manure incorporation

#### 3.3.1 Soil microbial community composition

**Figure 5:**
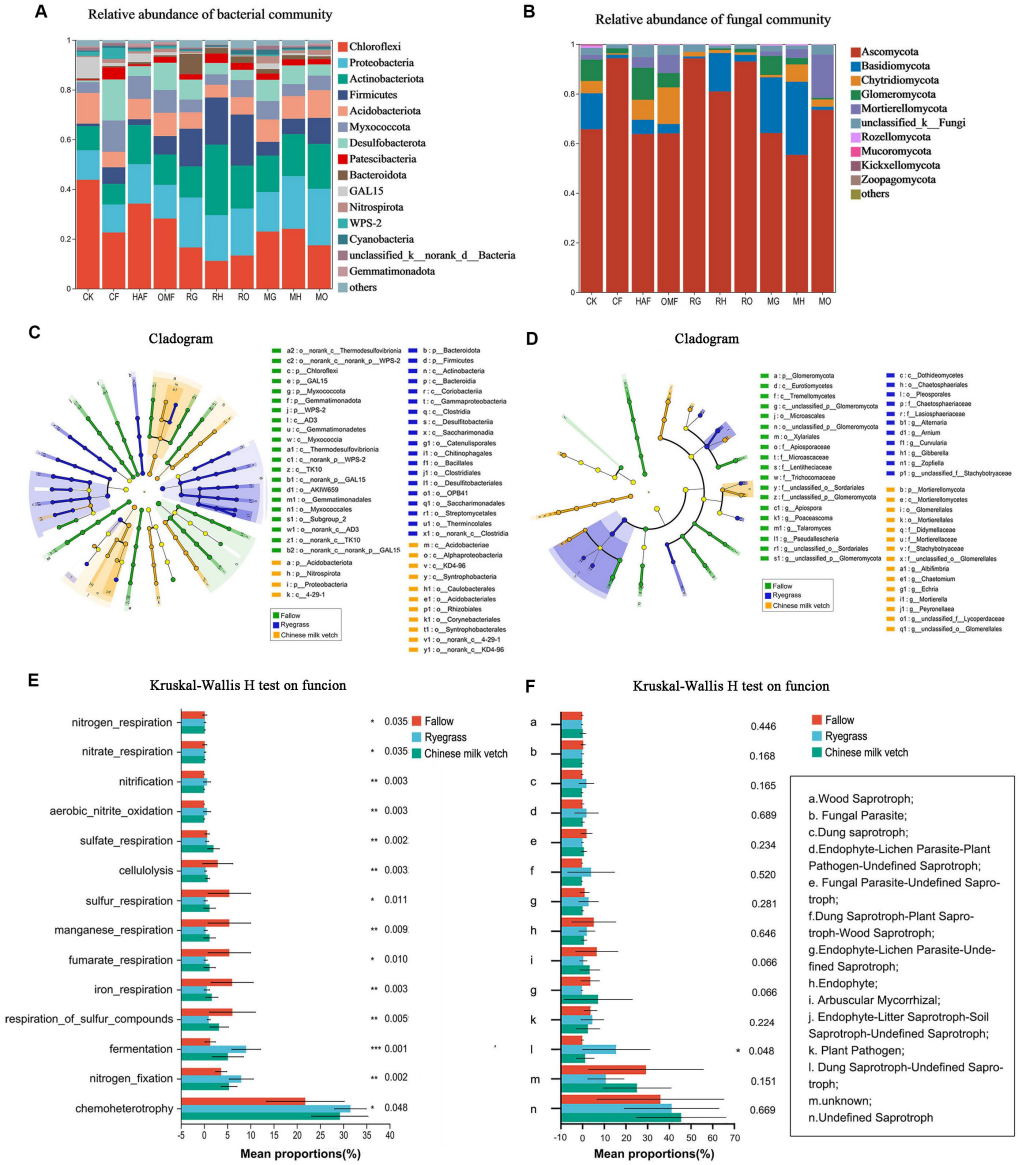
During the green manure incorporation period, the relative abundance of **(A)** bacterial community composition and **(B)** fungal community composition at phylum level; the LEfSe of **(C)** bacterial community and **(D)** fungal community regardless of the combined fertilizers; the functional prediction of **(E)** bacterial community by FAPROTAX and **(F)** fungal community by FUNGuild analysis regardless of the combined fertilizers. The *, **, and *** indicate the *P* < 0.05, *P* < 0.01, and *P* < 0.001 (Kruskal-Wallis H test), respectively.

We detected a total of 4,365 bacterial OTUs and 2,162 fungal OTUs from soil and identified to 43 bacterial phyla and 12 fungal phyla after green manure incorporation. The dominant bacterial phyla included *Chloroflexi* (11.30%–44.23%), *Proteobacteria* (11.35%–22.72%), *Actinobacteria* (8.34%–27.95%), *Firmicutes* (1.02%–10.63%), *Acidobacteria* (5.25%–11.89%), *Desulfobacteriota* (0.77–16.39%), *Myxococcota* (4.21%–12.77%), *Patescibacteria* (0.51–4.89%), *Bacteroidota* (0.41%–8.07%), GAL-15 (0.10%–8.63%), and *Nitrospirota* (0.29%–2.28%), which accounted for 97% of the total bacterial community (Figure 5A). Ryegrass incorporation significantly increased the relative abundance of Firmicutes in all three fertilizer combinations, compared with the fallow treatments (Supplementary Figure S3). Both ryegrass and Chinese milk vetch incorporation significantly increased the relative abundance of *Bacteroidota* under mineral fertilizer and *Patescibacteria* under humic acid fertilizer (Supplementary Figure S3). The fungal phylum *Ascomycota* was dominant, representing 55.37%–94.35% of the fungi in all treatments, followed by *Basidiomycota*, *Chytridiomycota*, and *Glomeromycota* (Figure 5B). Compared with the OMF treatment, RO significantly increased the relative abundance of *Ascomycota*, in which the class *Sordariomycetes* was significantly higher in RO. Also, the relative abundance of *Glomeromycota* was significantly higher in MG than in *CF* and RG (*p* = 0.039), while it was lower in MH and RH than in HAF (*p* = 0.027; Supplementary Figure S4).

According to the LEfSe (LDA > 3.5), the bacterial orders *Bacillales* and *Clostridiale* were significantly impacted by ryegrass incorporation, and the order *Rhizobiales* was more affected in Chinese milk vetch treatments (Figure 5C). For the fungal community, the classes *Dothideomycetes* and *Mortierellomycetes* were significantly impacted by ryegrass and Chinese milk vetch, respectively (Figure 5D).

#### 3.3.2 Soil microbial alpha diversity

In terms of the alpha diversity of the soil bacterial community during green manure incorporation, both the Chao1 and the Shannon indices exhibited significantly higher values in the green manure incorporation treatments compared to the fallow treatments, except for the Chao 1 in the RH and MO treatments (Table 1). Compared to the fallow treatments, the Chao1 exhibited increase ranging from 23.7 to 38.8% due to the green manure incorporation, while the Shannon index showed a range of increases from 7.0% to 14.7%. The RO treatment showed the greatest improvement in the Chao1 and Shannon indices among the 10 treatments (Table 1).

The Chao1 of the fungal communities in RG and MG treatments were significantly increased by 77.0% and 90.0%, respectively, compared to the CF treatment. Whereas there was no significant difference between the green manure incorporation treatments and the fallow treatments when combined with humic acid fertilizer or organic manure. The Shannon index was significantly decreased in MH and MO by 29.6% and 30.1% compared to HAF and OMF, respectively (Table 1). Additionally, there was no significant difference observed in the alpha diversity of the soil fungal community among the three combined fertilizer treatments for each green manure species.

#### 3.3.3 Soil microbial community structure and functional prediction

Based on the abundances of bacterial OTUs, the PCoA indicated that samples were clustered significantly according to the different green manure treatments (ANOSIM: R = 0.524, *p* = 0.001), and the samples from the organic manure treatments were separated from others (ANOSIM: R = 0.502, *p* = 0.001; Supplementary Figure S5A). For the fungal community, there were significant differences among all samples (ANOSIM: R = 0.617, *p* = 0.001). Additionally, green manure incorporation (ANOSIM: R = 0.426, *p* = 0.001) and fertilizers (ANOSIM: R = 0.279, *p* = 0.001) weakly but significantly affected the soil fungal communities (Supplementary Figure S5B).

The FAPROTAX functional prediction showed that the dominant functions such as chemoheterotrophy, nitrogen fixation and fermentation were significantly improved by the green manure application, which were related to C and N cycling. However, the functions related to sulfur cycle, such as respiration of sulfur compounds and sulfur respiration, were decreased in the green manure treatments, except for the relative abundance of sulfate respiration in Chinese milk vetch treatments (Figure 5E). FUNGuild analysis found that the relative abundance of dung saprotroph was significantly higher in ryegrass than Chinese milk vetch and fallow treatments, while the opposite was true in arbuscular mycorrhizal and endophyte litter saprotroph-soil saprotroph (Figure 5F).

#### 3.3.4 The relationship between the microbial community and soil properties and enzyme activities.

**Figure 6:**
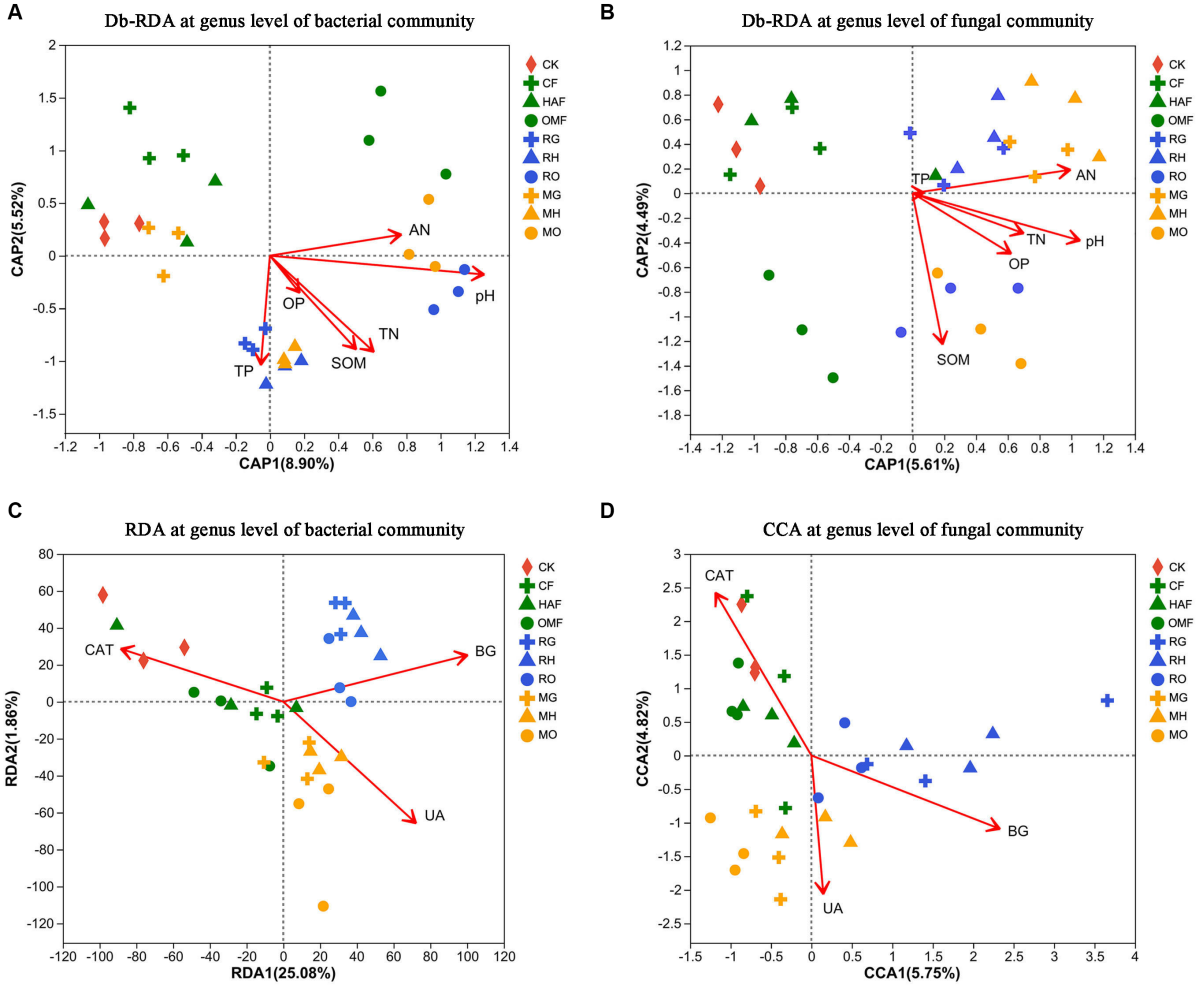
Db-RDA at genus level for the relationship **(A)** between soil bacterial communities and soil properties and **(B)** between the soil fungal communities and soil properties after green manure incorporation. The soil properties include pH, soil organic matter (SOM), total nitrogen content (TN), total phosphorus content (TP), Olsen-phosphorus (OP) and alkali-hydrolyzale nitrogen (AN). **(C)** RDA at genus level for the relationship between soil bacterial communities and soil enzyme activities (CAT, catalase; UA, urease; BG, *β*-glucosidase) after green manure incorporation; **(D)** CCA at genus level for correlation between the soil fungal communities and soil enzyme activities (CAT, catalase; UA, urease; BG, *β*-glucosidase) after green manure incorporation. Samples of CK are shown as red circles. The fallow treatments, ryegrass incorporation treatments and Chinese milk vetch incorporation treatments are shown as green, blue, and orange symbols, respectively. The combined mineral fertilizer, humic acid fertilizer and organic manure are indicated by triangle, diamond, and square, respectively.

The results of the db-RDA at the genus level showed that soil bacterial communities in green manure application were significantly correlated with the SOM and TN when combined with humic acid fertilizer and organic manure. The soil bacterial communities of RG, RH, and MH had a stronger positive correlation with TP, while soil from RO and MO correlated more positively with pH and AN (Figure 6A; Supplementary Table S3). While, the soil fungal communities were significantly correlated with the pH, SOM, AN, TN and OP (Supplementary Table S4). The soil fungal communities of OMF, RO, and MO showed a strong correlation with SOM, while samples from MG and MH were positively correlated with pH, AN, and TN (Figure 6B).

We also found that different soil enzyme activities had strong and unique correlations with both bacterial and fungal communities, varying among the different green manure treatments by using RDA and CCA (Figures 6C,D). In the fallow treatments, the soil bacterial and fungal communities were significantly positively correlated with catalase activity. Meanwhile, in the ryegrass and Chinese milk vetch treatments, the soil bacterial and fungal communities were significantly positively correlated with *β*-glucosidase activity and urease activity, respectively (Supplementary Tables S5, S6).

## 4 Discussion

Previous studies have reported that the incorporation of green manure with fertilizers affected soil physical and biological properties in long-term rice-green manure rotation systems (Chen et al., 2017). Our findings suggested that the utilization of green manure crops had a substantial influence on the soil microbial community throughout the growth and incorporation stages, especially the bacterial community. Additionally, we observed that these effects varied depending on the species of green manure crop applied and combined fertilizers.

### 4.1 Effects of green manure on soil microbial diversity and structure

Consistent with previous studies (He et al., 2020; Gao et al., 2021), the application of green manure resulted in a significant increase in bacterial diversity. However, our study also showed a contrasting trend in the diversity of the fungal community (see Table 1). The rhizosphere is a crucial area for nutrient exchange between plants and soil during green manure growth, which affects the microbial communities in this region (Bell et al., 2015). Previous studies have demonstrated that the diversity of the soil microbial community in the rhizosphere is influenced by both plant and soil properties (Lundberg et al., 2012; Mendes et al., 2014). In this study, the changes of bacterial and fungal diversities observed in rhizosphere soil may be attributed to the symbiotic and competitive interactions between green manure plants and microorganisms (Kuzyakov and Xu, 2013). For instance, green manure plant sediments serve as an effective and plant-specific source of organic matter for microbial growth in low-organic matter soil (Gmach et al., 2020; Saleem et al., 2020). However, green manure plants also obtain available nutrients from the soil to grow, which may form a competitive relationship with certain microbial groups in the rhizosphere soil. Furthermore, the effects of plant growth on rhizosphere microbial diversity were associated with soil nutrient conditions (Tedersoo et al., 2016). In our study, the bacterial diversity in the green manure rhizosphere significantly improved when combined with mineral fertilizer, while the effects of green manure on the diversity of rhizosphere microbes appeared to be less obvious when organic fertilizers were included, compared with the fallow soils.

Alterations in microbial diversity subsequent to the incorporation of green manure exhibited a congruent trend with the growth period, while the negative effect of green manure on fungal richness was mitigated (Table 1). Numerous of studies have suggested that the type and chemical composition of organic materials input to the soil affect the diversity of soil microorganisms (Khan et al., 2019; Liu et al., 2020). The microbial taxa that contribute to organic matter decomposition may benefit from the green manure residues (Wang et al., 2021). Fungal communities have been recognized as pivotal decomposers of organic materials, particularly with regard to cellulose and lignin in soil (Cox et al., 2001). Thus, the incorporation of green manure is expected to stimulate more fungal taxa in the degradation process. Sun et al. (2015) found that pig manure had a more positive effect on bacterial community diversity than other organic materials in a long-term green manure-rice system. The consistent findings were observed in our short-term experimental study, indicating that the incorporation of green manure with organic manure is advantageous for enhancing bacterial community diversity. Also, the highest diversity of bacterial community in RO could be linked to the more carbon and nitrogen provided by this combination and resulted in a wider range of ecological niches allowing for greater microbial growth in immature red soil. Consequently, the combination of green manure and organic manure may contribute to the augmentation of potential genetic diversity and the ability to resist environmental stress in immature red soil.

In addition, our findings indicated that the growth of green manure plants had a more significant impact on the bacterial community structure than fertilizer types in the rhizosphere soil. This was especially prominent in the case of ryegrass (as seen in Figure 3A), which had a strong root system. This may be attributed to the complex microenvironmental conditions within the rhizosphere, which confer a resistance to interference (Lovell et al., 2001). While the effects of green manure incorporation on the soil microbial community structures were related to both the green manure species and fertilizer types (see in Supplementary Figure S5).

### 4.2 Effects of green manure on soil microbial community composition

Different green manure species share common functional microbes, while can also make specific choices for microbes (Hartmann et al., 2009). In our study, both ryegrass and Chinese milk vetch growth significantly promoted the relative abundance of *Acidobacteria* compared to the fallow treatments. As typical oligotrophic taxa, the members of *Acidobacteria* were found to be advantageous to rhizosphere metabolism, and associated with carbon and nitrogen cycling (Fierer et al., 2007; Jiménez et al., 2012). The phylum *Firmicutes* and its class member *Clostridia* were markedly affected by ryegrass during both growth and incorporation periods (Figures 2C, 5C). *Firmicutes* has been reported to be beneficial for the carbon cycle due to its capacity to decompose plant-based polysaccharides (Pang et al., 2017), and the *Clostridia* can promote plant development and produce cellulase, which played a key role in degrading organic matter (Ueno et al., 2016; Abdallah et al., 2019). Also, the relative abundance of *Fibrobacteriota*, which have been reported to hydrolyse cellulose under anaerobic conditions, was significantly enhanced during the ryegrass incorporation when combined with organic manure (Supplementary Figure S3). In addition, fungal class *Sordariomycetes* and *Dothideomycetes* were significantly promoted by ryegrass application (Figures 2D, 5D), which are known to secrete a variety of cellulase, hemicellulose and lignocellulosic enzymes (Wang et al., 2021). Therefore, the promotion of these bacterial and fungal groups associated with organic material decomposition and carbon cycle demonstrates that the growth and incorporation of ryegrass are beneficial for improving carbon metabolism in the immature red soil. In the case of Chinese milk vetch, being a legume, it significantly increased the relative abundance of bacterial taxa associated with nitrogen cycling, such as phylum *Cyanobacteria* in rhizosphere soil and order *Rhizobiales* during both growth and incorporation periods (Supplementary Figure S1; Figures 2, 5). *Cyanobacteria* are widely recognized for their capacity to nitrogen-fixing and their potential for agricultural applications (Singh et al., 2014). The increased abundance of *Rhizobiales* in the rhizosphere soil of Chinese milk vetch is understandable, as it is known to form a symbiotic relationship with legume roots, and fixing nitrogen in nodules (Ferguson et al., 2018). However, the improvement observed during the incorporation of Chinese milk vetch is unexpected since the symbiosis does not occur beyond the roots. This may be attributed to the legacy effects (Jing and Cong, 2022), whereby *Rhizobiales* proliferates in the soil throughout the course of plant growth and eventually evolves into the beneficial group enhanced by Chinese milk vetch. Furthermore, the fungal phylum *Glomeromycota*, whose members are commonly known as arbuscular mycorrhizal fungi (Smith and Read, 2010), was apparently enhanced by Chinese milk vetch in combination with mineral fertilizer (Supplementary Figure S4). This enhancement implies potential advantages for organic matter decomposition and absorption of nutrients by subsequent crops, such as phosphorous and nitrogen (Hodge and Fitter, 2010; Cheng et al., 2012).

### 4.3 Effects of green manure on soil enzyme activities and microbial functions

Changes in the composition of the microbial community can be partially confirmed by the results of the enzyme activities and microbial functional prediction (Figures 3–5). The activity of soil enzymes, which are produced by various soil microorganisms, plays a central role in soil nutrient cycling and can sensitively reflect the decomposition of organic matter in soil (Sinsabaugh et al., 2008). We found that urease activity was more highly promoted under Chinese milk vetch incorporation, while *β*-glucosidase activity was higher under ryegrass incorporation. This result was consistent with findings made by Khan et al. (2019), who found that hydrolase activity in the carbon cycle was higher after non-legume (barley) incorporation, while the activity of soil enzymes involved in the nitrogen cycle was higher after legume (hairy vetch) incorporation. In line with the changes observed in soil enzyme activities, the structures of the soil microbial community and the activities of soil enzymes also exhibited a specific correlation associated with green manure (Figures 6C,D). The incorporation of ryegrass, which has a higher C: N ratio and cellulose content, significantly affected the bacterial and fungal communities correlated with *β*-glucosidase activity, which is key to organic matter cycling, especially carbon degradation (Bońska and Lasota, 2017). The microbial communities affected by Chinese milk vetch containing more nitrogen content had a strong correlation with the activity of urease, which is considered to be the main enzyme in the nitrogen cycle (Sinsabaugh et al., 2008).

Additionally, the increased relative abundances of microbial functions linked to organic carbon turnover, including chemoheterotrophy, fermentation, and dung saprotroph, confirmed the stimulation of organic carbon metabolism by the utilization of ryegrass. Meanwhile, the proportion of microbial functions involved in nitrogen metabolism was greater in the rhizosphere soil of Chinese milk vetch, suggesting that the rhizosphere activity of Chinese milk vetch promotes nitrogen cycling in immature red soil. The root secretions provide diverse compounds such as amino acids, organic acids and other secondary metabolites that stimulate the growth of various microorganisms under nutrient-limited immature soil and vary with the plant species (El Zahar et al., 2014). In addition, it is notable that the abundance of nitrogen cycle-related functions, such as nitrogen fixation, aerobic nitrite oxidation and nitrification, were also significantly promoted by ryegrass incorporation (Figure 5E), consisting with a previous study in which Italian ryegrass residues promote the growth of microbes involved in the N cycle (He et al., 2020). This may be due to the specific compounds from ryegrass residues that can alter the soil properties and promote the growth of species associated with nitrogen metabolism in the immature soil. In a study by Yan et al. (2018), it was observed that litter from different plant species, with their distinct components, selectively stimulated specific bacteria with diverse biological functions, such as the production of specific extracellular enzymes and promoting community succession. According to our results, we conjectured that the green manure species affected specific soil microbial taxa that could produce different extracellular enzymes and functions, consequently affecting the organic matter degradation and nutrient cycling in immature red soil. Nonetheless, additional investigations are required to gain a deeper understanding.

### 4.4 Effects of green manure on soil physical properties and microbial growth

The application of green manures combined fertilizers led to changes in soil conditions and thus influenced the bioavailability of nutrients beneficial to microbial growth. Consistent with previous studies suggesting that green manure can improve soil physical and chemical properties (Ma et al., 2021), our results indicated that the application of green manure significantly increased soil pH and nitrogen levels, particularly nitrogen availability, in immature red soil (as shown in Table 2). The improvement in soil pH may be attributed to the alkaline nature of green manure, which neutralizes the protons in acidic soil (Rukshana et al., 2013). Surprisingly, the levels of available nitrogen (AN) in the RG and RO treatments were greatly enhanced, surpassing those in the MG and MO treatments. Previous studies have suggested that non-leguminous cover crops enhance nutrient availability by reducing nutrient leaching (Doltra et al., 2019). However, the ability of leguminous plants to fix nitrogen results in higher nitrogen content in their tissues and thus releases more available nitrogen during degradation (Mathesius, 2022). Although the higher AN content in the ryegrass application aligns with our results in bacterial functional prediction (Figure 5E), further investigation into the specific mechanisms underlying this observation is warranted.

Previously, the input of organic materials has been shown to stimulate the growth of soil microorganisms, so as to improve the soil microbial biomass (Ge et al., 2010). In this study, the incorporation of green manure significantly improved SMBC, with the MO treatment giving the highest increase (Figure 4A). Compared to ryegrass, Chinese milk vetch has a lower fiber content and higher protein content, which is more easily immobilized by soil microorganisms (Watthier et al., 2020). And the organic manure contained more available nutrients for microorganisms, thus the MO combination has an advantage in promoting the growth of microorganisms in immature red soil. It is also noteworthy that the addition of humic acid significantly reduced the SMBC, although humic acid significantly increased the SOC (Figure 4A; Table 2). This finding is consistent with a previous study that observed humic acid application reduced SMBC when combined with biochar (Holatko et al., 2020). It could possibly be attributed to the inhibition of microbial assimilation due to the fact that the organic carbon present in humic acid is stable and difficult to decompose.

### 4.5 Relationship between the soil microbial community and soil properties regulated by green manure application

Studies have suggested that the soil microbial community was mainly determined by soil properties such as pH, soil nutrients, and organic matter content (Rinklebe and Langer, 2006; Yu et al., 2019). In this study, we found that the improvements of pH, SOM, AN and TN were the key environmental factors affecting both soil bacterial and fungal communities when green manure application combined with organic fertilizers (Figures 6A,B; Supplementary Tables S3, S4). As demonstrated in a previous study, the observed increase in soil total nitrogen (TN) and soil organic matter (SOM) can, in part, be attributed to the rise in the number of potential functional genes following green manure application. This increase is primarily driven by bacteria taxa that are involved in the decomposition process (Zheng et al., 2018b). Our results confirmed the positive correlation between key factors including pH, TN, AN and SOM and the bacterial taxa associated with carbon and nitrogen cycling (e.g., *Firmicutes*, *Fibrobacterota*, and *Bacteroidota*), which were significantly increased following green manure application (Supplementary Figures S3, S6). Also, the significant increase in soil pH under green manure application may related to the notable increase in soil sulfate respiration function, indicating green manuring may be a potential practice in mitigating acidification of immature red soil (Gao et al., 2021). Additionally, the relationships between the soil properties and microbial communities varied with the types of combined fertilizers. Soil pH and soil organic matter positively affected bacterial and fungal community variations, respectively, when combined with organic manure. While, soil total P and alkali-hydrolyzale N were the important variables affecting the soil bacterial community and fungal community, respectively, when combined with humic acid (Figures 6A,B). The mechanism underlying these effects remains unclear, but it is likely to be associated with the physical structure, composition, and bioactive substances of the two different organic fertilizers.

## 5 Conclusion

In the present study, we investigated the effects of various green manure-fertilizer combinations on soil microbial community characteristics in low-fertility immature red soil. The application of green manure had a positive effect on soil pH, nitrogen availability, soil microbial growth, activity, bacterial diversity and specific microbial groups involved in soil carbon and nitrogen cycles, such as *Firmicutes*, *Acidobacteria*, and *Glomeromycota*. The significant correlation between soil microbial community and soil properties highlights the crucial role of green manure in stimulating soil nutrient cycling by altering the microbial community, leading to the improvement of soil efficient fertility and ecosystem functioning. Furthermore, the impact of green manure on soil microbial communities and functions exhibited plant species specificity, and were also influenced by the types of combined fertilizers, pointing out the need for targeted management strategies that account for both the type of green manure crops and the specific fertilizer requirements of the soil. In conclusion, our study emphasizes the potential of green manure as a sustainable agricultural practice for improving soil biological health and underscores the importance of considering the role of soil microbes in regulating ecosystem function. However, further research is necessary to determine how these alterations of soil microbial community contribute to the subsequent crops.

## Data availability statement

The datasets presented in this study can be found in online repositories. The names of the repository/repositories and accession number(s) can be found in the article/Supplementary material.

## Author contributions

JX: Formal Analysis, Funding acquisition, Methodology, Visualization, Writing – original draft. LS: Data curation, Funding acquisition, Investigation, Writing – review & editing. XZ: Conceptualization, Validation, Visualization, Writing – review & editing. KC: Investigation, Supervision, Writing – review & editing. JW: Conceptualization, Funding acquisition, Project administration, Resources, Writing – review & editing.

## Footnotes

^1^http://huttenhower.sph.harvard.edu/lefse

^2^http://www.loucalab.com/archive/FAPROTAX/

^3^https://github.com/UMNFuN/FUNGuild
