# Supplementary material for: Various green manure-fertilizer combinations affect the soil microbial community and function in immature red soil
Source: Front Microbiol. 2023 Dec 14;14:1255056. doi: 10.3389/fmicb.2023.1255056 (PMC10757628; doi:10.3389/fmicb.2023.1255056)
Supplement: Supplementary file 1 [file Data_Sheet_1.docx]

**

Supplementary materials**

**Fig. S1** Multiple groups comparison of relative abundance of bacterial taxa in the fallow and green manure crops in growth period. CF: fallow with mineral fertilizer; RG: ryegrass combined with mineral fertilizer; HAF: fallow with humic acid fertilizer; RH: ryegrass combined with humic acid fertilizer; OMF: fallow with organic manure; RO: ryegrass combined with organic manure; MG: Chinese milk vetch combined with mineral fertilizer; MH: Chinese milk vetch combined with humic acid fertilizer; MO: Chinese milk vetch combined with organic manure.


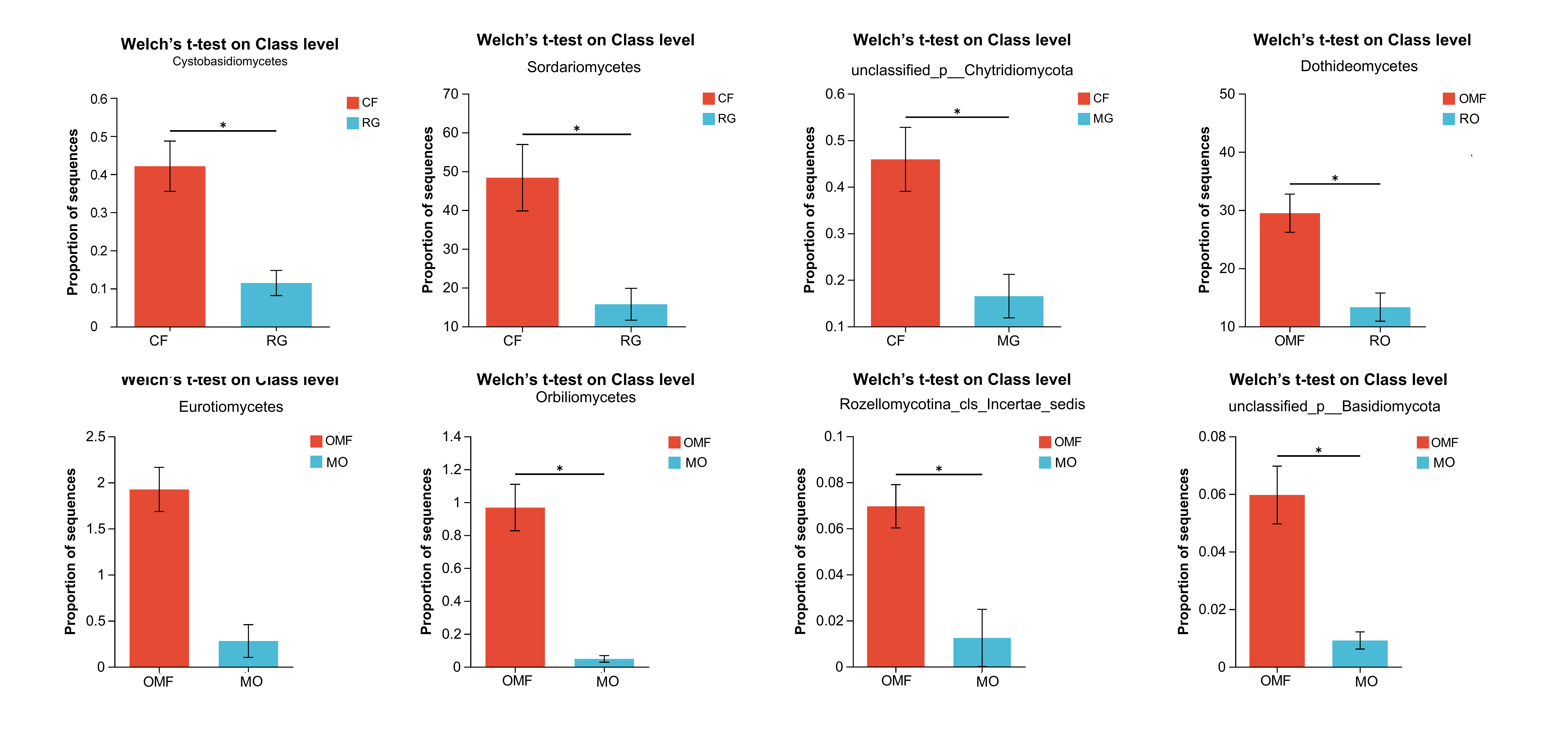
**Fig. S2** Multiple groups comparison of relative abundance of fungal taxa in the fallow and green manure crops in growth period. CF: fallow with mineral fertilizer; RG: ryegrass combined with mineral fertilizer; HAF: fallow with humic acid fertilizer; RH: ryegrass combined with humic acid fertilizer; OMF: fallow with organic manure; RO: ryegrass combined with organic manure; MG: Chinese milk vetch combined with mineral fertilizer; MH: Chinese milk vetch combined with humic acid fertilizer; MO: Chinese milk vetch combined with organic manure.

**

**

**Fig. S3** Multiple groups comparison of relative abundance of bacterial taxa in the fallow and green manure crops in incorporation period. CF: fallow with mineral fertilizer; RG: ryegrass combined with mineral fertilizer; HAF: fallow with humic acid fertilizer; RH: ryegrass combined with humic acid fertilizer; OMF: fallow with organic manure; RO: ryegrass combined with organic manure; MG: Chinese milk vetch combined with mineral fertilizer; MH: Chinese milk vetch combined with humic acid fertilizer; MO: Chinese milk vetch combined with organic manure.


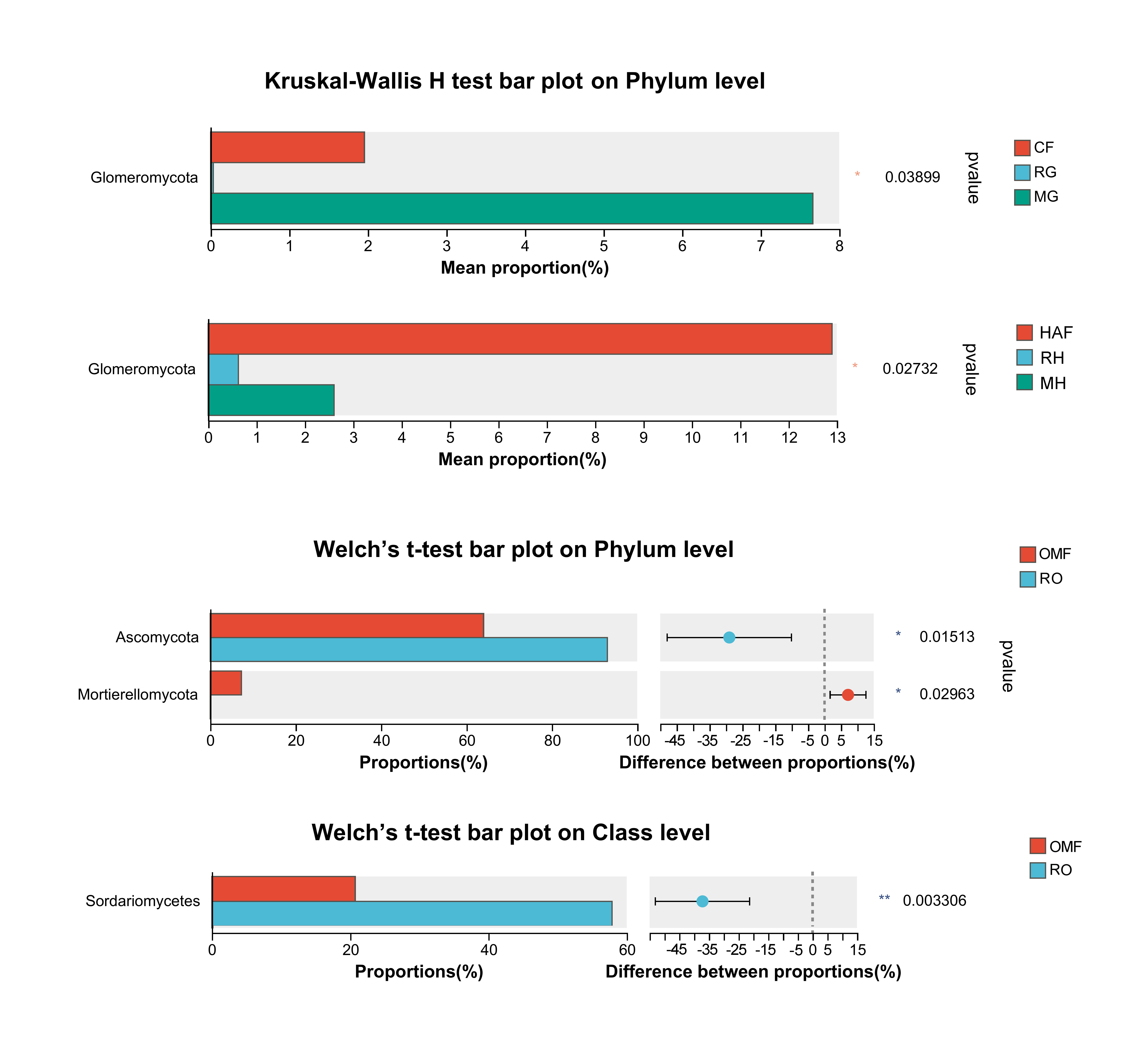


**Fig. S4** Multiple groups comparison of relative abundance of fungal taxa on phylum and class level in the fallow and green manure crops in incorporation period. CF: fallow with mineral fertilizer; RG: ryegrass combined with mineral fertilizer; HAF: fallow with humic acid fertilizer; RH: ryegrass combined with humic acid fertilizer; OMF: fallow with organic manure; RO: ryegrass combined with organic manure; MG: Chinese milk vetch combined with mineral fertilizer; MH: Chinese milk vetch combined with humic acid fertilizer.


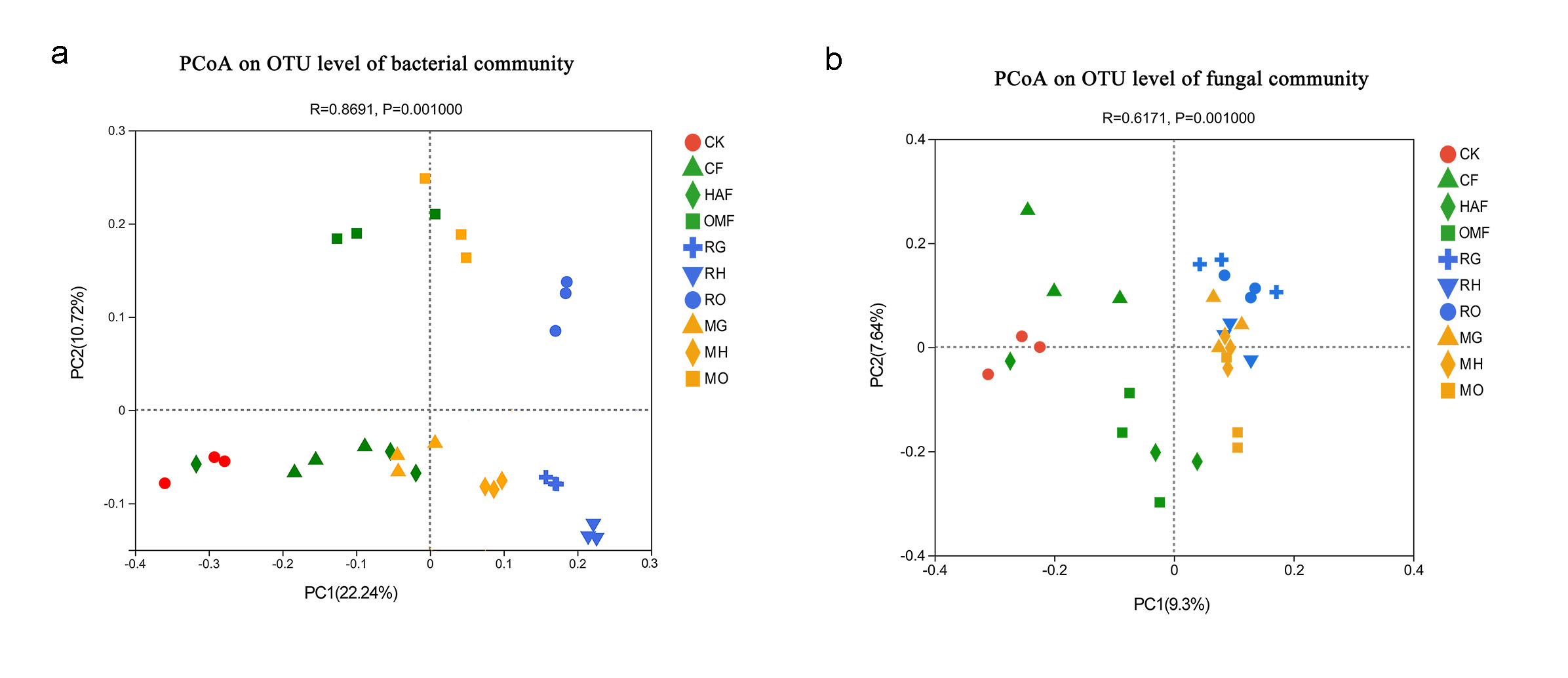
**Fig. S5** PCoA at OTU level of a) soil bacterial community and b) fungal community after green manure incorporation. Samples of CK were showed as red circle. The fallow treatments, ryegrass incorporation treatments and Chinese milk vetch incorporation treatments were showed as green, blue and orange symbols, respectively.

**
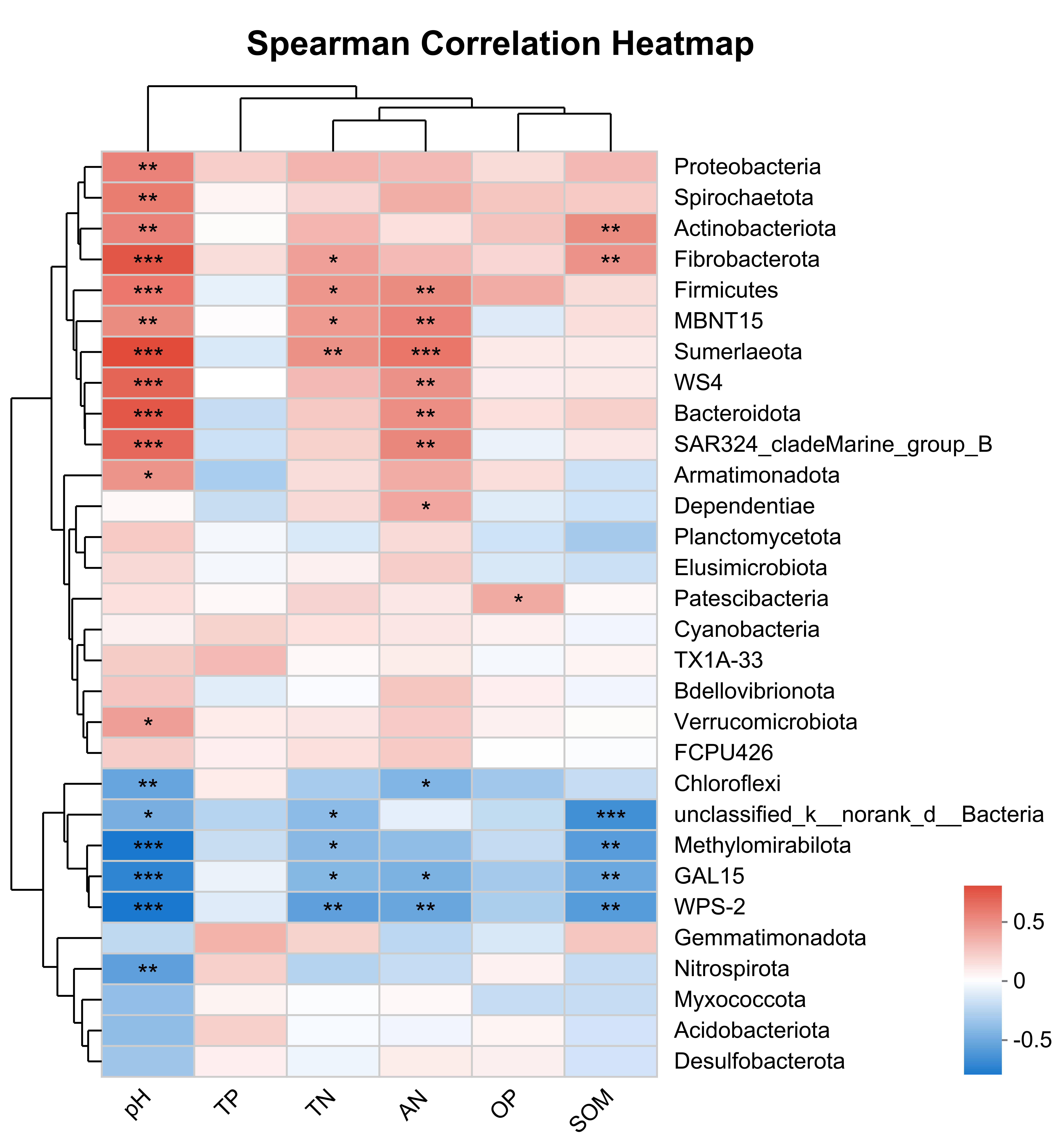
**

**Fig. S6** Spearman correlation heatmap based on the bacterial community and environmental variables. The X and Y axes of the thermal graph are the environmental factors and species (at phylum level), respectively, and the R and P values are calculated. R values are shown in different colors in the graph, and the right color card is the color partition of the different R values. *, **, and *** indicate that means are significantly different at P <0.05, <0.01, and <0.001, respectively.

**Table S1** The nutritional content of different organic fertilizers used in the study.

| Soil properties | Humic acid fertilizer | Commercial organic fertilizer |
| --- | --- | --- |
| pH | 6.15 | 7.43 |
| Organic matter (g kg^-1^) | 753.94 | 291.97 |
| Total N (g kg^-1^) | 9.03 | 14.38 |
| Total P (g kg^-1^) | 7.87 | 17.92 |
| Total K (g kg^-1^) | 1.3 | 33 |
| Price (RMB t^-1^) | 1200 | 1000 |

**Table S2** Results of the analysis of effects by green manure incorporation and fertilizer addition on soil properties using general linear model.

| Soil properties | SS | | |  | Green manure | | |  | Fertilizer | | |
| --- | --- | --- | --- | --- | --- | --- | --- | --- | --- | --- | --- |
|  | F |  | P |  | F |  | P |  | F |  | P |
| pH | 25.861 |  | **P<0.001** |  | 77.188 |  | **<0.001** |  | 23.128 |  | **<0.001** |
| Soil organic matter (SOM) | 69.065 |  | **P<0.001** |  | 1.536 |  | >0.05 |  | 273.789 |  | **<0.001** |
| Total nitrogen content (TN) | 12.959 |  | **P<0.001** |  | 2.564 |  | >0.05 |  | 42.591 |  | **<0.001** |
| Alkali-hydrolyzale nitrogen (AN) | 7.180 |  | **P<0.001** |  | 19.249 |  | **<0.001** |  | 5.221 |  | **<0.05** |
| Total phosphorus content (TP) | 2.080 |  | P>0.05 |  | 3.282 |  | >0.05 |  | 4.202 |  | **<0.05** |
| Olsen-phosphorus (OP) | 1.204 |  | P>0.05 |  | 2.406 |  | >0.05 |  | 0.606 |  | >0.05 |

**Table S3** Results of correlation and significance for db-RDA on bacterial community and soil properties.

| Soil properties | CAP1 | CAP2 | r^2^ | P values |
| --- | --- | --- | --- | --- |
| **pH** | **0.9997** | **0.023** | **0.7952** | **0.001** |
| **TN** | **0.6029** | **-0.7978** | **0.446** | **0.002** |
| **TP** | **-0.2268** | **-0.9739** | **0.4339** | **0.001** |
| OP | 0.445 | -0.8956 | 0.0503 | 0.505 |
| **AN** | **0.9496** | **0.3133** | **0.3362** | **0.003** |
| **SOM** | **0.5172** | **-0.8559** | **0.3825** | **0.002** |

**Table S4** Results of correlation and significance for db-RDA on fungal community and soil properties.

| Soil properties | CAP1 | CAP2 | r^2^ | P values |
| --- | --- | --- | --- | --- |
| **pH** | **0.9324** | **-0.3613** | **0.6077** | **0.001** |
| **TN** | **0.8964** | **-0.4432** | **0.2819** | **0.02** |
| TP | 0.9981 | 0.062 | 0.0012 | 0.983 |
| **OP** | **0.7665** | **-0.6422** | **0.2997** | **0.006** |
| **AN** | **0.9804** | **0.1968** | **0.4887** | **0.001** |
| **SOM** | **0.1459** | **-0.9893** | **0.7768** | **0.001** |

**Table S5** Results of correlation and significance for RDA on bacterial community and soil enzyme activities.

| Enzyme activities | RDA1 | RDA2 | r^2^ | P values |
| --- | --- | --- | --- | --- |
| UA | 0.734 | -0.6791 | 0.4377 | 0.001 |
| CAT | -0.9896 | 0.1442 | 0.4271 | 0.002 |
| BG | 0.9134 | 0.4071 | 0.6598 | 0.001 |

**Table S6** Results of correlation and significance for CCA on fungal community and soil enzyme activities.

| Enzyme activities | RDA1 | RDA2 | r^2^ | P values |
| --- | --- | --- | --- | --- |
| UA | 0.0583 | -0.9983 | 0.4394 | 0.001 |
| CAT | -0.4446 | 0.8958 | 0.7558 | 0.001 |
| BG | 0.9154 | -0.4024 | 0.7074 | 0.001 |
